# A colorimetric assay for vanillin detection by determination of the luminescence of o-toluidine condensates

**DOI:** 10.1371/journal.pone.0194010

**Published:** 2018-04-20

**Authors:** Jin Zhao, Haixiong Xia, Tingyu Yu, Lu Jin, Xuehua Li, Yinghui Zhang, Liping Shu, Lingwen Zeng, Zhixu He

**Affiliations:** 1 Guizhou Provincial Key Laboratory for Regenerative Medicine, Tissue Engineering and Stem Cell Research Center, Department of Immunology, School of Basic Medicine, Guizhou Medical University, Guiyang, China; 2 Affiliated Hospital of Guizhou Medical University, State Key Laboratory for Medicinal Plant Efficacy and Utilization, Guizhou Medical University, Guiyang, China; 3 Second Clinical Medical College, Guangzhou University of Chinese Medicine, Guangzhou, China; 4 School of Food Science and Engineering, Foshan University, Foshan, Guangdong Province, China; 5 Key Laboratory of Regenerative Biology, South China Institute for Stem Cell Biology and Regenerative Medicine, Guangzhou Institutes of Biomedicine and Health, Chinese Academy of Sciences, Guangzhou, China; Waseda University, JAPAN

## Abstract

Vanillin (4-hydroxy-3-methoxybenzaldehyde), a food additive with rich milk flavor, is commonly used in the food, beverage and cosmetic industries. However, excessive consumption of vanillin may cause liver and kidney damage. Therefore, methods for detecting and controlling the level of vanillin in food, especially in infant powder, have important practical significance. In this study, we established a colorimetric assay for vanillin detection. The detection was performed under high-temperature and acidic conditions, which can induce the reaction of the aldehyde group of vanillin with the amino group of o-toluidine. The resulting product had a maximum absorption at 363 nm, which was quantified by a UV spectrophotometer. This assay had a limit of detection (*LOD*) of 1 pg mL^−1^ and a linear range between 1 μg mL^−1^ and 100 μg mL^−1^. The average recoveries at three spiked levels were in the range from 91.1% to 101.6% with a relative standard deviation (*RSD*) of 4.62% ~ 7.27%.

## Introduction

Vanillin (4-hydroxy-3-methoxybenzaldehyde) is one of the most widely used flavoring agents in the food, beverage and cosmetic industries[1]. It is a white needle-like crystalline powder with an intensely sweet taste similar to creamy vanilla[2]. Natural vanillin is obtained from the orchid Vanilla planifolia and its bean[3]. Only a small amount of the vanillin used in foods and beverages is derived from the natural plant because of its high cost. It was reported that plant-derived vanillin accounted for only 0.2% of the market requirement[4]. Most of the vanillin produced was obtained through artificial synthesis[5] or biotechnological production[6, 7] and was further used for the manufacture of numerous household products, deodorants, air fresheners, floor polishes and herbicides. According to the regulations of the FDA, the concentration of vanillin in food should not exceed 70 mg kg^-1^. China National Food Safety Standard (GB-2760-2011)[8] requires that the amount of vanillin used in the preparation of vanilla, chocolate, or butter is in the range from 25% to 30% and that the amount directly used in biscuits and cakes is in the range from 0.1% to 0.4%. It also forbids the usage of vanillin in formula for 0–6-month-old infants due to the potential health problems induced by excess ingestion, including impaired liver, kidney, and spleen functions[9, 10]. Therefore, vanillin should be monitored due to its food safety concerns and potential environmental pollution effects.

There are several methods for vanillin detection, such as liquid chromatography-mass spectrometry (LC-MS)[11, 12], capillary electrophoresis (CE)[13], electrochemical methods[14–16], high-performance liquid chromatography (HPLC)[17], and gas chromatography-mass spectrometry (GC-MS)[18]. In the past ten years, LC-MS has been widely used for the determination of vanillin[12, 19, 20] but has suffered from a few disadvantages, such as its complicated and time-consuming nature and poor recovery. Common electrochemical methods include oscillographic polarography[21, 22], the ion-selective electrode method[23, 24], and the chemically modified electrode method[25]. Therefore, methods that provide useful information, high sensitivity, and good resolution and selectivity for vanillin determination are needed.

**Fig 1:**
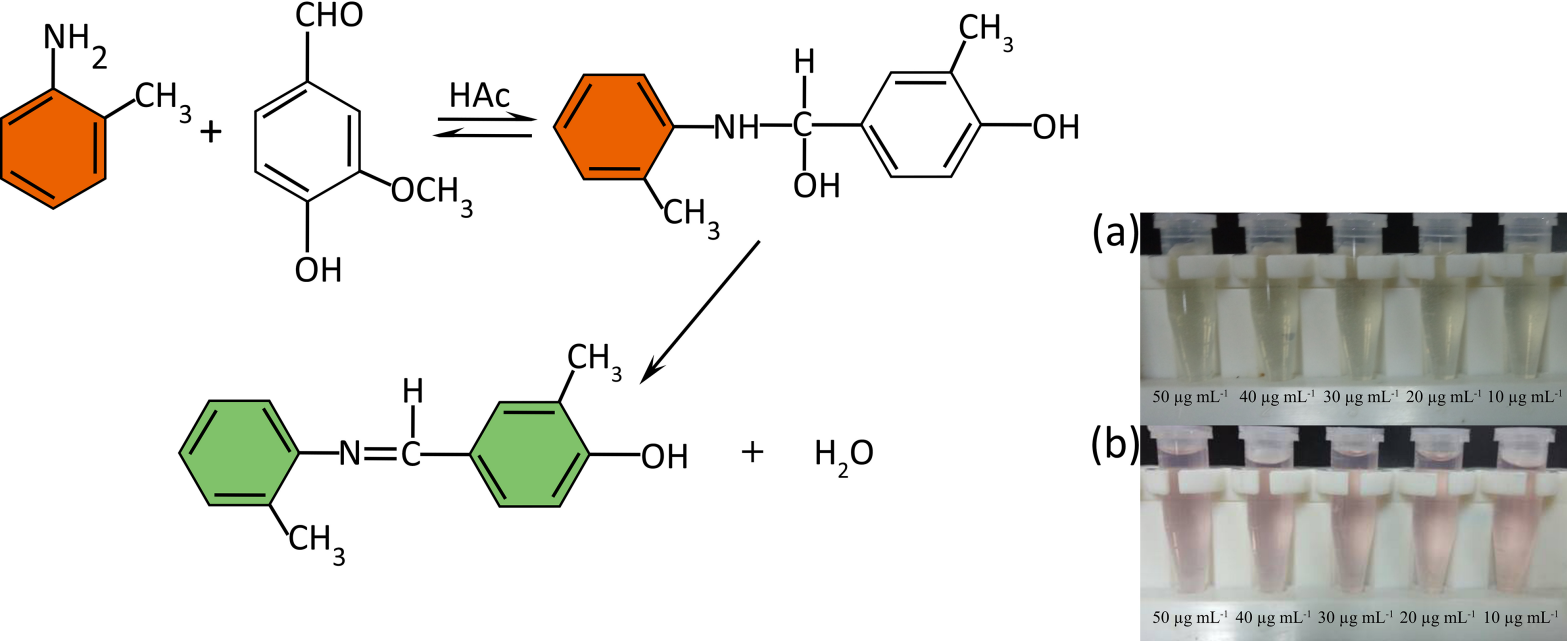
Schematic illustration of the chemical structures of o-toluidine and vanillin and the condensation reaction between the two compounds. (a) A light-green color is visible before heating, and (b) a reddish-brown color is visible after heating.

In this paper, a simple colorimetric assay was developed for fast and sensitive vanillin detection. Vanillin contains a carboxy group and an aldehyde group, the latter of which can participate in a condensation reaction with the amine group of o-toluidine under high-temperature and acidic conditions to form an imine group (C = N) (Fig 1). The absorbance of the condensation product, which is highest at 363 nm, was determined by a UV spectrophotometer. The condensation product has a light-green visible light waves at 538 nm. The method was optimized and used to determine vanillin, and it can be applied to the determination of vanillin in real samples such as milk powder extracts.

## Materials and methods

### Reagents and chemicals

Vanillin was purchased Shanghai Sangon Biological Engineering Technology (Shanghai, China). o-Toluidine and N, N-dimethylformamide were purchased from Shanghai Aladdin Bio-Chem Technology Co., LTD (Shanghai, China). Sodium citrate tribasic dihydrate and Tris were purchased from Sigma-Aldrich (Steinheim, Germany). Glycine was purchased from GenStar Biosolutions Co., Ltd. (Beijing, China). Citric acid, disodium hydrogen, HCl, NaOH, Na_2_HPO_4_ and acetic acid were purchased from Guangzhou Chemical Reagent Factory (Guangzhou, China). Acetonitrile was purchased from Tianjin Damao Chemical Reagent Factory (Tianjin, China). n-Hexane was purchased from Chengdu Kelong Chemical Reagent Factory (Chengdu, China). Other chemicals were purchased from standard commercial sources and were of analytical grade purity.

### Instrumentation

A Cary 100 UV spectrophotometer (Varian, America) equipped with a 1 cm quartz cell was used to measure the vanillin standard samples. A microplate reader (BioTek Epoch, America) was used to determine the light absorbance. Water from a Milli-Q purification system (18.2 MΩ/cm, Millipore, Billerica, MA, USA) was used in all experiments.

### Preparation of standard solutions

A standard stock solution of 1 mg mL^−1^ vanillin was prepared by dissolution in water. o-Toluidine solution was prepared by mixing o-toluidine with N, N-dimethylformamide at 1:3 (v/v). The ranges of pH of four different acid buffers about 2–3 were prepared: glycine-HCl buffer (25% 0.2 M glycine, 22% 0.2 M HCl), Na_2_HPO_4_-citric acid buffer (3.2% 0.2 M Na_2_HPO_4_, 84.8% 0.1 M citric acid), citric acid-sodium citrate tribasic dihydrate buffer (7.5% 0.2 M citric acid, 92.5% 0.3 M sodium citrate tribasic dihydrate) and 0.2 M HCl buffer. All the stock standard solutions were stored at 4°C in the dark.

### Preparation of samples

A standard stock solution of 1 mg mL^−1^ vanillin was diluted to 500 μg mL^−1^, 400 μg mL^−1^, 300 μg mL^−1^, 200 μg mL^−1^,100 μg mL^−1^, 75 μg mL^−1^, 50 μg mL^−1^, 25 μg mL^−1^, 1 μg mL^−1^, 100 ng mL^−1^, 10 ng mL^−1^, 1 ng mL^−1^, 100 pg mL^−1^, 10 pg mL^−1^ and 1 pg mL^−1^. A milk powder sample was prepared for real sample detection. One gram of milk powder was added into a 15 mL centrifuge tube and dissolved with 3.0 mL of Tris buffer. Acetonitrile (7 mL) then was added into this milk powder solution and mixed by vigorously vortexing for 1 min. The mixture was centrifuged at 10,000 rpm at room temperature for 10 min[26]. The supernatant was transferred to another 15 mL centrifuge tube, and 2 mL of n-hexane was added to the supernatant. The mixture was vortexed for 1 min and then centrifuged at 10,000 rpm at room temperature for 5 min. The supernatant was taken and filtered through a 0.22 μm nylon membrane and stored at 4°C in the dark.

### Experimental design

Vanillin standard samples from 1 pg mL^−1^ to 500 μg mL^−1^ were tested in this experiment. Briefly, 1 mL of vanillin solution and 200 μL of o-toluidine were added to a 1.5 mL Eppendorf tube, and the mixture was shaken for 5 min at room temperature. Acetic acid solution (200 μL) was then added to the mixture, and the tube was placed in a boiling water bath for 15 min at 100°C. After the tube cooled to room temperature, the absorbance was measured by a UV spectrophotometer. The same method was used to detect vanillin in milk powder extract samples that were prepared previously.

### Data analysis

Dose-response curve fitting and data analysis were performed using linear regression analysis with Origin Pro software (version 17.0, Hampton, Massachusetts, USA). Each result is presented as the mean ± SD. To detect statistical correlations, Spearman’s rank correlation coefficient (R^2^) was calculated between the absorbance and vanillin concentration over a wide range of vanillin concentrations.

## Results and discussion

### Optimization of reaction parameters

**Fig 2:**
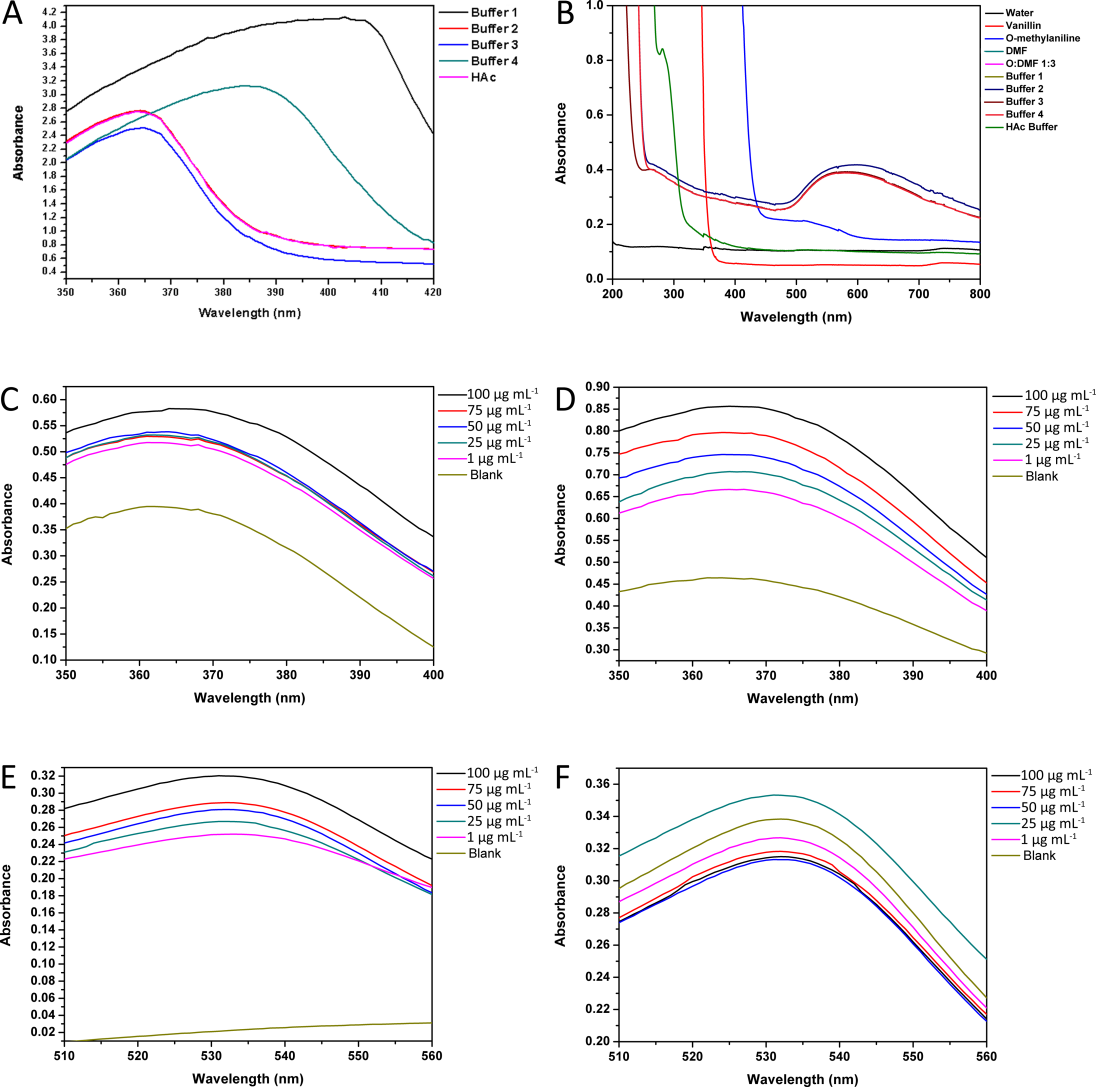
Absorbance spectra obtained by the UV spectrophotometer. (A) Spectra of four kinds of acid buffer, (B) spectra of all reagents. (C, E) spectra of standard samples from 1 μg mL^−1^ to 100 μg mL^−1^ before heating, (D, F) the absorption spectra of standard samples from 1 μg mL^−1^ to 100 μg mL^−1^ after heating.

To select the acid buffer, we tested four different kinds: Na_2_HPO_4_-citric acid buffer, glycine-HCl buffer, citric acid-sodium citrate tribasic dihydrate buffer, and HCl buffer, which showed maximum absorption values at 403 nm, 363 nm, 363 nm, 384 nm, respectively (Fig 2A, S1 Table). The UV-vis absorption curve showed that Na_2_HPO_4_-citric acid buffer and acetic acid buffer provided almost the same results. However, o-toluidine had poor solubility in these four acid buffers: a visible amount of o-toluidine was observed floating on the buffer solutions. Only acetic acid buffer could dissolve o-toluidine. In addition, all the reagents involved in the reaction were evaluated, and no interference was found (Fig 2B, S2 Table).

Four kinds of acid buffers were mixed with vanillin and o-toluidine solution. All the samples with different acid buffers visibly appeared light green before heating in a bath, and the intensity of the color depended on the concentration of vanillin. In contrast to the solutions mixed with the other buffers, the solution mixed with acetic acid buffer changed to a reddish-brown color after heating (Fig 1). This reddish-brown color appeared after heating because the o-toluidine that did not react with vanillin, only o-toluidine underwent an oxidation reaction with water after heating. Therefore, we compared the changes in color before and after heating. The results showed that we could distinguish the light green color intensity of the mixed solutions with our naked eyes before heating. Moreover, the absorbance of the samples at 538 nm, as determined by a UV spectrophotometer, was observed to decrease when the vanillin concentration was reduced from 1 μg mL^−1^ to 100 μg mL^−1^ before heating (Fig 2E, S5 Table). However, the reaction solution changed into reddish-brown color after heating cause the o-toluidine underwent an oxidation reaction with water, it interfered the green blue light wavelengths range so that the color intensity after heating could not be distinguished by the naked eye, and the absorbance at 538 nm of the solutions was disrupted and showed irregularity, thus a maximum absorption wave in 25 μg mL^-1^. (Fig 2F, S6 Table). Therefore, the results obtained by the UV spectrophotometer showed that the absorption peak of vanillin and o-toluidine reaction products remained at 363 nm. After heating, the absorbance from 350 nm to 400 nm increased, which demonstrated that vanillin and o-toluidine reacted more completely with heating than without heating (Fig 2C and 2D, S3 and S4 Tables). All the UV-vis absorption curve data from S1 to S6 Tables were matched to Fig 1A to 1F.

To investigate the effect of pH on the reaction, the assay was carried out using 500 μL of vanillin standard (50 μg mL^−1^) solution and 100 μL of o-toluidine. Volumes of 1 M HCl solution from 20 μL to 240 μL were added into 12 tubes, resulting in solutions with pH ranging from 7 to 3 (Table 1). The result showed that vanillin could not react with o-toluidine at neutral pH. The absorption of the condensation product was highest at pH 4–5 but then decreased under more acidic conditions (pH<4). This demonstrated that the optimum pH for the vanillin and o-toluidine reaction was under weakly acidic conditions.

**Table 1:** The absorbance and pH of 50 μg mL^−1^ vanillin standard samples.

50 μg mL^-1^ Vanillin (μL)	20	40	60	80	100	120	140	160	180	200	220	240
**Absorbance**	0.313	0.364	0.393	0.418	0.477	0.501	0.521	0.516	0.477	0.462	0.456	0.435
**pH**	7	7	6–7	6	5–6	5–6	5	4–5	4–5	4	3–4	3

### Vanillin standard samples characterized by UV spectrophotometry

**Fig 3:**
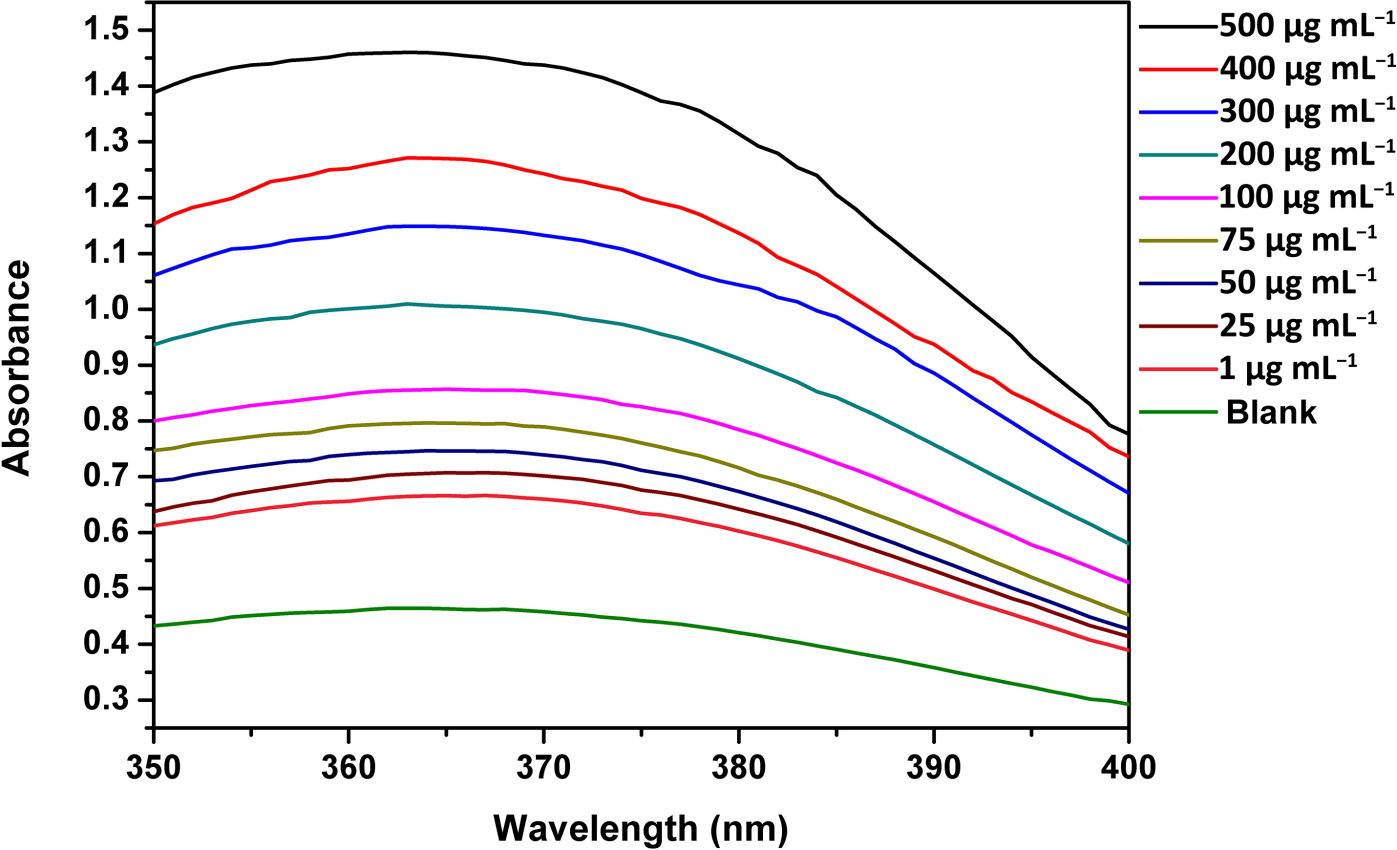
Absorption spectra of standard vanillin samples obtained by the UV spectrophotometer. The vanillin concentration ranged from 1 μg mL^−1^ to 500 μg mL^−1^. The absorption spectra of the vanillin samples showed that as the vanillin concentration decreased, the absorbance declined.

The results showed that the condensation product had absorption from 350 nm to 390 nm with a maximum absorption at 363 nm. The absorbance of the samples as determined by a UV spectrophotometer, was observed to decrease when the vanillin concentration was reduced from 500 μg mL^−1^ to 1 μg mL^−1^ (Fig 3, S7 Table). Water mixed with o-toluidine solution was used as a blank for comparison, and the absorbance of the standard samples was much higher than that of the blank. According to the previous literature[27], vanillin solution showed an absorption peak at 348 nm, but in our study, the product absorption peak was at 363 nm. This may be because a spatial steric hindrance is produced by the two benzene rings after vanillin reacted with o-toluidine and because the combination of these two benzene rings was quite stable. After vanillin reacted with o-toluidine, the solution visibly appeared light green.

### Determination of vanillin standard samples by microplate reader

**Fig 4:**
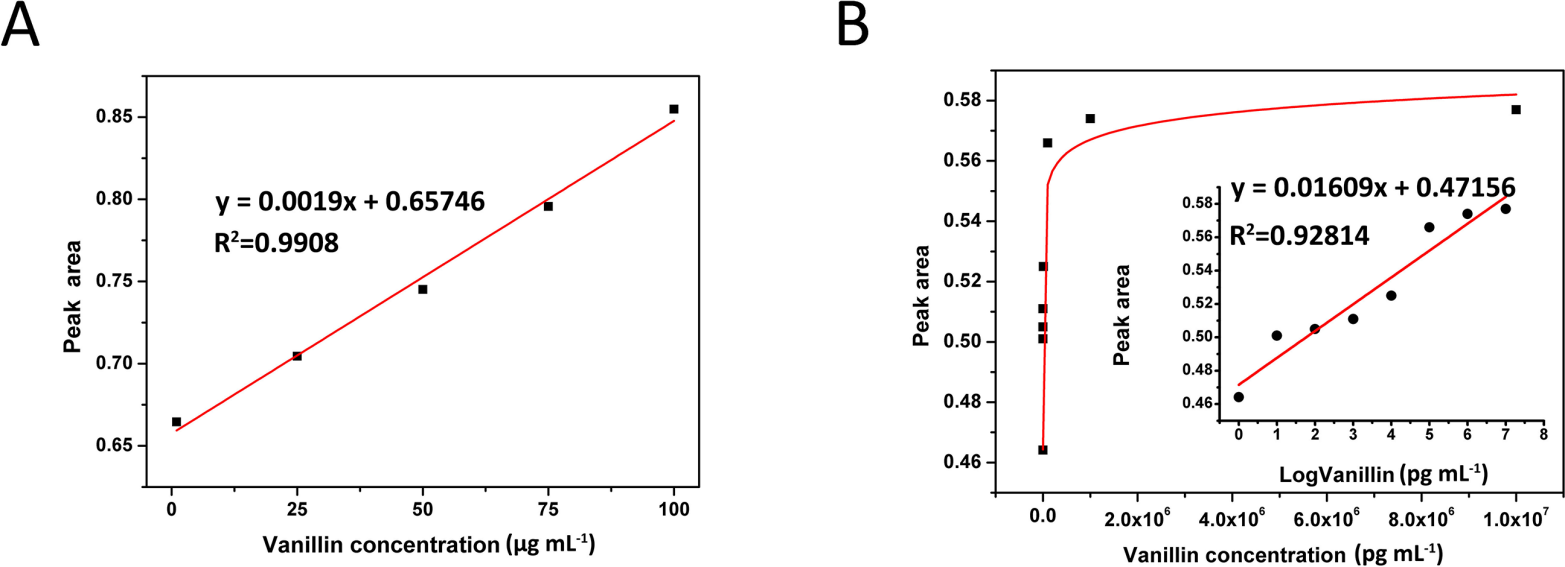
Correlation analysis between vanillin concentration and absorbance. (A) Correlation between vanillin concentration and absorbance from 1 μg mL^-1^ to 100 μg mL^-1^ (R^2^ = 0.9908). (B) R^2^ = 0.92814 from blank to 10^7^ pg mL^-1^.

For faster multiplex testing, we used a microplate reader to detect vanillin standard samples at 368 nm. The results showed that as the concentration of the vanillin standard samples decreased, the absorbance also declined (Table 2). Strong correlations were found between the absorbance and vanillin concentration over a wide range of vanillin concentrations (Fig 4). A strong correlation was observed (R^2^ = 0.9908) from 1 μg mL^−1^ to 100 μg mL^−1^ vanillin (Fig 4A). In addition, the absorbance was also dependent on the vanillin concentration from 1 μg mL^−1^ to 1 pg mL^−1^ (Fig 4B, R^2^ = 0.92814).

**Table 2:** Absorbance of standard vanillin samples.

Vanillin (pg mL^-1^)	10^7^	10^6^	10^5^	10^4^	10^3^	10^2^	10^1^	10^0^	Blank
**Absorbance**	0.697	0.662	0.658	0.652	0.605	0.598	0.593	0.577	0.547

According to the correlation analysis, the equation used for the quantification of vanillin at concentrations from 1 μg mL^-1^ to 100 μg mL^-1^ is shown in Eq (1), and the equation used for vanillin at concentrations from 0 to 10^7^ pg mL^-1^ is shown in Eq (2).

$$A_{a}368=0.0019Ca+0.65746$$ (1)

$$A_{b}368=0.01609log10Cb+0.47156$$ (2)

*C*_*a*_, The vanillin concentration from 100 μg mL^-1^ to 1 μg mL^-1^

*C*_*b*_, The vanillin concentration from 10^7^ pg mL^-1^ to blank

### Colorimetric detection for vanillin spiked samples

**Fig 5:**
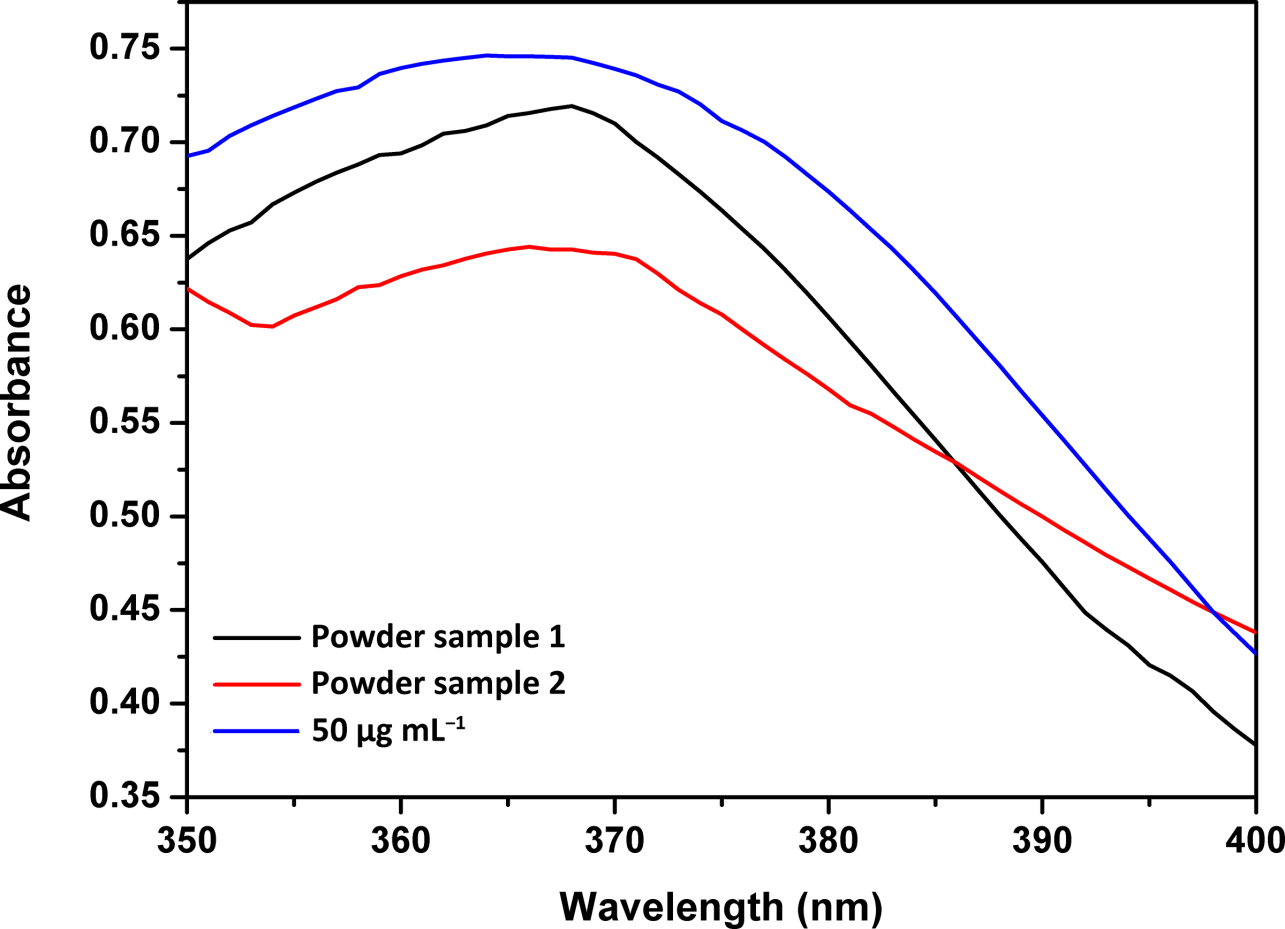
Milk powder samples compared with vanillin standard sample (50 μg mL^-1^).

In this study, two brands of milk powder were detected. The present assay could also effectively detect vanillin in spiked samples. The results showed that the milk powder samples had the same maximum absorption at 363 nm as the 50 μg mL^-1^ vanillin standard sample and that the absorbance of the milk powder samples was lower than that of the vanillin standard samples (Fig 5, S8 Table). Moreover, the concentration of vanillin in both milk powders did not exceed the Chinese national standard (5 mg 100 mL^-1^).

### Recovery rates of the colorimetric assay for vanillin detection

The practical application of the colorimetric assay was demonstrated by its application to detect vanillin in milk powder samples. Recovery experiments were performed by spiking different amounts of vanillin into milk powder samples (S9 Table). Acceptable recovery rates in the spiked milk powder samples (between 91.1% and 101.6%) for vanillin detection were obtained, which confirmed that the proposed colorimetric assay was able to detect vanillin in spiked milk powder samples with little interference (Table 3).

**Table 3:** Measurements of vanillin spiked in milk powder samples (n = 6).

Sample	Spiking level (vanillin μg mL^-1^)	Found	Recovery (%)	RSD (%)
**Milk powder**	1000	1280±77.68	120.23	6.07
	500	560±25.87	106.83	4.62
	10	9.82±0.71	91.1	7.27

Although several novel and promising methods, such as LC-MS, HPLC, and GC-MS methods, have been developed for vanillin detection in food, beverages, chocolate, milk powder and other types of samples, they still require specialized equipment and well-trained personnel. The present assay is characterized by simplified handling and a decreased need for laboratory instruments and has a short sample preparation time. It has a limit of detection (*LOD*) of 1 pg mL^−1^ and can analyze milk powder samples.

The colorimetric assay we established presents several advantages over other methods. First, it is easy to operate with simple equipment. Compared with the chemically modified electrode method, the electrochemical sensor technique is ideal for the determination of vanillin due to its advantages of cheap instrumentation, small sample volume and rapid analysis. However, there is a major obstacle encountered in the detection of vanillin contamination with a bare electrode, namely, a high overpotential, which causes poor reproducibility, selectivity and sensitivity. The colorimetric assay is based on the light-green fluorescence product and requires only a microplate reader to detect the absorption at 363 nm. This colorimetric assay has advantages such as speed, efficiency, reproducibility, small sample volume, low solvent consumption and ease of contaminant removal.

Second, the sample pretreatment method for this colorimetric assay is simpler than that for LC-MS methods. Sample preparation is the most important step in the development of methods for the analysis of real samples. LC-MS methods, which are widely used to determine vanillin, have advantages in terms of sensitivity and specificity but are time consuming and require tedious operation. In a study on milk powder analysis, sample preparation took over 1 hour[26]. HPLC remains the most dominant technique for the analysis of vanillin, but such methods also have disadvantages, such as the considerable time required for column equilibration[17]. Therefore, an extraction technique should be rapid, simple and inexpensive. In this study, tedious vanillin extraction was avoided.

Third, the time of the present assay is relatively shorter than that of current methods. In contrast, GC-MS took more than 3 hours for vanillin detection and required highly trained personnel because of its complex operating steps[28]. The colorimetric assay for vanillin determination can be completed within 30 min.

## Conclusion

Compared with other methods, the present colorimetric assay had a great advantage in terms of sample pretreatment. The time of the whole sample preparation process was no more than 20 min. The *LOD* of this assay was 1 pg mL^−1^, which is much lower than the Chinese national standard (5 mg 100 mL^-1^).

## Supporting information

S1 Table The UV-vis absorption curve data of Fig 2A. Spectra of four kinds of acid buffer.
(DOCX)

S2 Table The UV-vis absorption curve data of Fig 2B. Spectra of all reagents.
(DOCX)

S3 Table The UV-vis absorption curve data of Fig 2C. Spectra of standard samples from 1 μg mL^−1^ to 100 μg mL^−1^ before heating.
(DOCX)

S4 Table The UV-vis absorption curve data of Fig 2D. Spectra of standard samples from 1 μg mL^−1^ to 100 μg mL^−1^ after heating.
(DOCX)

S5 Table The UV-vis absorption curve data of Fig 2E. Spectra of standard samples from 1 μg mL^−1^ to 100 μg mL^−1^ before heating.
(DOCX)

S6 Table The UV-vis absorption curve data of Fig 2F. Spectra of standard samples from 1 μg mL^−1^ to 100 μg mL^−1^ after heating.
(DOCX)

S7 Table The UV-vis absorption curve data of Fig 3. The absorption spectra of the vanillin samples from 1 μg mL^−1^ to 500 μg mL^−1^.
(DOCX)

S8 Table The UV-vis absorption curve data of Fig 5. Milk powder samples compared with vanillin standard sample (50 μg mL^-1^).
(DOCX)

S9 Table Measurements details data of vanillin spiked in milk powder samples (n = 6). (DOCX)
