# Supplementary material for: A colorimetric assay for vanillin detection by determination of the luminescence of o-toluidine condensates
Source: PLoS One. 2018 Apr 20;13(4):e0194010. doi: 10.1371/journal.pone.0194010 (PMC5909897; doi:10.1371/journal.pone.0194010)
Supplement: S1 Table — Spectra of four kinds of acid buffer. (DOCX) [file pone.0194010.s001.docx]

**S1 table. The UV-vis absorption curve data of Fig. 2 A.** Spectra of four kinds of acid buffer.

| **Wavelength**  **(nm)** | **The Absorbance of different acid buffer** | | | | |
| --- | --- | --- | --- | --- | --- |
|  | **Buffer 1** | **Buffer 2** | **Buffer 3** | **Buffer 4** | **HAc** |
| **420** | 2.42 | 0.74 | 0.52 | 0.83 | 0.74 |
| **419** | 2.53 | 0.74 | 0.52 | 0.87 | 0.74 |
| **418** | 2.66 | 0.74 | 0.52 | 0.87 | 0.74 |
| **417** | 2.80 | 0.74 | 0.53 | 0.92 | 0.74 |
| **416** | 2.94 | 0.74 | 0.53 | 0.97 | 0.74 |
| **415** | 3.10 | 0.74 | 0.53 | 1.03 | 0.74 |
| **414** | 3.26 | 0.75 | 0.53 | 1.09 | 0.74 |
| **413** | 3.42 | 0.75 | 0.54 | 1.14 | 0.75 |
| **412** | 3.57 | 0.75 | 0.54 | 1.20 | 0.75 |
| **411** | 3.72 | 0.75 | 0.54 | 1.27 | 0.75 |
| **410** | 3.86 | 0.75 | 0.54 | 1.34 | 0.75 |
| **409** | 3.95 | 0.75 | 0.55 | 1.43 | 0.75 |
| **408** | 4.03 | 0.76 | 0.55 | 1.51 | 0.76 |
| **407** | 4.08 | 0.76 | 0.55 | 1.59 | 0.76 |
| **406** | 4.08 | 0.76 | 0.55 | 1.68 | 0.76 |
| **405** | 4.09 | 0.77 | 0.56 | 1.74 | 0.76 |
| **404** | 4.11 | 0.77 | 0.56 | 1.84 | 0.77 |
| **403** | 4.13 | 0.75 | 0.57 | 1.94 | 0.77 |
| **402** | 4.11 | 0.78 | 0.57 | 2.02 | 0.78 |
| **401** | 4.11 | 0.78 | 0.58 | 2.12 | 0.76 |
| **400** | 4.10 | 0.79 | 0.58 | 2.23 | 0.79 |
| **399** | 4.10 | 0.80 | 0.59 | 2.34 | 0.79 |
| **398** | 4.10 | 0.80 | 0.60 | 2.43 | 0.80 |
| **397** | 4.10 | 0.81 | 0.61 | 2.53 | 0.81 |
| **396** | 4.09 | 0.82 | 0.62 | 2.63 | 0.82 |
| **395** | 4.08 | 0.84 | 0.63 | 2.72 | 0.83 |
| **394** | 4.09 | 0.85 | 0.65 | 2.79 | 0.85 |
| **393** | 4.07 | 0.87 | 0.66 | 2.87 | 0.86 |
| **392** | 4.06 | 0.89 | 0.68 | 2.93 | 0.88 |
| **391** | 4.05 | 0.91 | 0.70 | 2.99 | 0.90 |
| **390** | 4.05 | 0.93 | 0.73 | 3.03 | 0.93 |
| **389** | 4.03 | 0.96 | 0.76 | 3.06 | 0.95 |
| **388** | 4.03 | 0.97 | 0.79 | 3.09 | 0.98 |
| **387** | 4.02 | 0.99 | 0.82 | 3.11 | 0.98 |
| **386** | 3.99 | 1.03 | 0.87 | 3.12 | 1.02 |
| **385** | 3.99 | 1.08 | 0.91 | 3.12 | 1.07 |
| **384** | 3.96 | 1.13 | 0.94 | 3.13 | 1.12 |
| **383** | 3.95 | 1.18 | 0.99 | 3.13 | 1.17 |
| **382** | 3.92 | 1.24 | 1.05 | 3.12 | 1.23 |
| **381** | 3.90 | 1.31 | 1.13 | 3.11 | 1.30 |
| **380** | 3.89 | 1.40 | 1.19 | 3.10 | 1.38 |
| **379** | 3.86 | 1.48 | 1.28 | 3.07 | 1.47 |
| **378** | 3.82 | 1.58 | 1.38 | 3.06 | 1.57 |
| **377** | 3.83 | 1.68 | 1.48 | 3.04 | 1.65 |
| **376** | 3.79 | 1.79 | 1.59 | 3.03 | 1.79 |
| **375** | 3.75 | 1.91 | 1.70 | 3.00 | 1.90 |
| **374** | 3.72 | 1.99 | 1.82 | 2.97 | 1.99 |
| **373** | 3.69 | 2.12 | 1.93 | 2.94 | 2.10 |
| **372** | 3.65 | 2.23 | 2.03 | 2.92 | 2.22 |
| **371** | 3.63 | 2.34 | 2.14 | 2.89 | 2.33 |
| **370** | 3.58 | 2.45 | 2.24 | 2.85 | 2.44 |
| **369** | 3.55 | 2.55 | 2.33 | 2.82 | 2.53 |
| **368** | 3.51 | 2.65 | 2.42 | 2.79 | 2.64 |
| **367** | 3.48 | 2.69 | 2.46 | 2.76 | 2.68 |
| **366** | 3.44 | 2.73 | 2.49 | 2.72 | 2.72 |
| **365** | 3.41 | 2.76 | 2.51 | 2.69 | 2.74 |
| **364** | 3.37 | 2.76 | 2.51 | 2.66 | 2.75 |
| **363** | 3.33 | 2.76 | 2.50 | 2.62 | 2.75 |
| **362** | 3.30 | 2.75 | 2.49 | 2.58 | 2.73 |
| **361** | 3.25 | 2.73 | 2.47 | 2.54 | 2.71 |
| **360** | 3.21 | 2.70 | 2.44 | 2.50 | 2.68 |
| **359** | 3.17 | 2.68 | 2.41 | 2.46 | 2.66 |
| **358** | 3.14 | 2.65 | 2.38 | 2.43 | 2.63 |
| **357** | 3.10 | 2.62 | 2.35 | 2.39 | 2.60 |
| **356** | 3.05 | 2.58 | 2.31 | 2.35 | 2.56 |
| **355** | 3.01 | 2.54 | 2.27 | 2.30 | 2.52 |
| **354** | 2.96 | 2.50 | 2.23 | 2.25 | 2.48 |
| **353** | 2.91 | 2.45 | 2.18 | 2.20 | 2.43 |
| **352** | 2.86 | 2.41 | 2.13 | 2.15 | 2.39 |
| **351** | 2.80 | 2.37 | 2.09 | 2.10 | 2.34 |
| **350** | 2.76 | 2.32 | 2.05 | 2.06 | 2.30 |
