# Supplementary material for: A colorimetric assay for vanillin detection by determination of the luminescence of o-toluidine condensates
Source: PLoS One. 2018 Apr 20;13(4):e0194010. doi: 10.1371/journal.pone.0194010 (PMC5909897; doi:10.1371/journal.pone.0194010)
Supplement: S2 Table — Spectra of all reagents. (DOCX) [file pone.0194010.s002.docx]

**S2 table. The UV-vis absorption curve data of Fig. 2 B.** Spectra of all reagents.

| **Wavelength (nm)** | **The Absorbance of different** **all the reagents** | | | | | | | | | |
| --- | --- | --- | --- | --- | --- | --- | --- | --- | --- | --- |
|  | **Water** | **Vanillin** | **O-methylaniline** | **DMF** | **O:DMF 1:3** | **Buffer 1** | **Buffer 2** | **Buffer 3** | **Buffer 4** | **HAc** |
| **420** | 0.11 | 0.05 | 0.61 | 10.00 | 0.11 | 1.05 | 0.29 | 0.27 | 0.27 | 0.11 |
| **419** | 0.11 | 0.05 | 0.65 | 10.00 | 0.11 | 1.05 | 0.29 | 0.27 | 0.27 | 0.11 |
| **418** | 0.11 | 0.05 | 0.69 | 10.00 | 0.11 | 1.06 | 0.29 | 0.27 | 0.27 | 0.11 |
| **417** | 0.11 | 0.06 | 0.74 | 10.00 | 0.11 | 1.06 | 0.29 | 0.27 | 0.27 | 0.11 |
| **416** | 0.11 | 0.06 | 0.80 | 10.00 | 0.11 | 1.06 | 0.29 | 0.27 | 0.27 | 0.11 |
| **415** | 0.11 | 0.06 | 0.86 | 10.00 | 0.11 | 1.06 | 0.29 | 0.27 | 0.27 | 0.11 |
| **414** | 0.11 | 0.06 | 0.92 | 10.00 | 0.11 | 1.06 | 0.29 | 0.27 | 0.27 | 0.11 |
| **413** | 0.11 | 0.06 | 0.95 | 10.00 | 0.11 | 1.06 | 0.30 | 0.28 | 0.27 | 0.11 |
| **412** | 0.11 | 0.06 | 1.02 | 10.00 | 0.11 | 1.06 | 0.30 | 0.28 | 0.27 | 0.11 |
| **411** | 0.11 | 0.06 | 1.10 | 10.00 | 0.11 | 1.06 | 0.30 | 0.28 | 0.27 | 0.11 |
| **410** | 0.11 | 0.06 | 1.18 | 10.00 | 0.12 | 1.07 | 0.30 | 0.28 | 0.27 | 0.12 |
| **409** | 0.11 | 0.06 | 1.25 | 10.00 | 0.12 | 1.07 | 0.30 | 0.28 | 0.27 | 0.12 |
| **408** | 0.11 | 0.06 | 1.35 | 10.00 | 0.12 | 1.07 | 0.30 | 0.28 | 0.28 | 0.12 |
| **407** | 0.11 | 0.06 | 1.45 | 10.00 | 0.12 | 1.07 | 0.30 | 0.28 | 0.28 | 0.12 |
| **406** | 0.11 | 0.06 | 1.57 | 10.00 | 0.12 | 1.07 | 0.30 | 0.28 | 0.28 | 0.12 |
| **405** | 0.11 | 0.06 | 1.70 | 10.00 | 0.12 | 1.08 | 0.30 | 0.28 | 0.28 | 0.12 |
| **404** | 0.11 | 0.06 | 1.84 | 10.00 | 0.12 | 1.08 | 0.30 | 0.28 | 0.28 | 0.12 |
| **403** | 0.11 | 0.06 | 1.97 | 10.00 | 0.12 | 1.08 | 0.30 | 0.28 | 0.28 | 0.12 |
| **402** | 0.11 | 0.06 | 2.14 | 10.00 | 0.12 | 1.06 | 0.30 | 0.28 | 0.28 | 0.12 |
| **401** | 0.11 | 0.06 | 2.40 | 10.00 | 0.12 | 1.07 | 0.30 | 0.28 | 0.28 | 0.12 |
| **400** | 0.11 | 0.06 | 2.81 | 10.00 | 0.12 | 1.07 | 0.30 | 0.28 | 0.28 | 0.12 |
| **399** | 0.11 | 0.06 | 10.00 | 10.00 | 0.12 | 1.08 | 0.30 | 0.28 | 0.28 | 0.12 |
| **398** | 0.11 | 0.06 | 10.00 | 10.00 | 0.12 | 1.12 | 0.30 | 0.28 | 0.28 | 0.12 |
| **397** | 0.11 | 0.06 | 10.00 | 10.00 | 0.12 | 1.09 | 0.30 | 0.28 | 0.28 | 0.12 |
| **396** | 0.11 | 0.06 | 10.00 | 10.00 | 0.12 | 1.09 | 0.30 | 0.28 | 0.28 | 0.12 |
| **395** | 0.11 | 0.06 | 10.00 | 10.00 | 0.12 | 1.09 | 0.30 | 0.28 | 0.28 | 0.12 |
| **394** | 0.11 | 0.06 | 10.00 | 10.00 | 0.12 | 1.10 | 0.30 | 0.28 | 0.28 | 0.12 |
| **393** | 0.11 | 0.06 | 10.00 | 10.00 | 0.12 | 1.10 | 0.30 | 0.28 | 0.28 | 0.12 |
| **392** | 0.11 | 0.06 | 10.00 | 10.00 | 0.12 | 1.10 | 0.30 | 0.28 | 0.28 | 0.12 |
| **391** | 0.11 | 0.06 | 10.00 | 10.00 | 0.13 | 1.10 | 0.30 | 0.28 | 0.28 | 0.13 |
| **390** | 0.11 | 0.06 | 10.00 | 10.00 | 0.13 | 1.10 | 0.30 | 0.28 | 0.28 | 0.13 |
| **389** | 0.11 | 0.06 | 10.00 | 10.00 | 0.13 | 1.11 | 0.30 | 0.28 | 0.28 | 0.13 |
| **388** | 0.11 | 0.06 | 10.00 | 10.00 | 0.13 | 1.11 | 0.30 | 0.28 | 0.28 | 0.13 |
| **387** | 0.11 | 0.06 | 10.00 | 10.00 | 0.13 | 1.11 | 0.30 | 0.28 | 0.29 | 0.13 |
| **386** | 0.11 | 0.06 | 10.00 | 10.00 | 0.13 | 1.11 | 0.31 | 0.29 | 0.29 | 0.13 |
| **385** | 0.11 | 0.06 | 10.00 | 10.00 | 0.13 | 1.11 | 0.31 | 0.29 | 0.29 | 0.13 |
| **384** | 0.11 | 0.06 | 10.00 | 10.00 | 0.13 | 1.12 | 0.31 | 0.29 | 0.29 | 0.13 |
| **383** | 0.11 | 0.06 | 10.00 | 10.00 | 0.13 | 1.12 | 0.31 | 0.29 | 0.29 | 0.13 |
| **382** | 0.11 | 0.06 | 10.00 | 10.00 | 0.13 | 1.12 | 0.31 | 0.29 | 0.29 | 0.13 |
| **381** | 0.11 | 0.06 | 10.00 | 10.00 | 0.13 | 1.12 | 0.31 | 0.29 | 0.29 | 0.13 |
| **380** | 0.11 | 0.06 | 10.00 | 10.00 | 0.13 | 1.12 | 0.31 | 0.29 | 0.29 | 0.13 |
| **379** | 0.11 | 0.06 | 10.00 | 10.00 | 0.13 | 1.11 | 0.31 | 0.29 | 0.29 | 0.13 |
| **378** | 0.11 | 0.06 | 10.00 | 10.00 | 0.13 | 1.11 | 0.31 | 0.29 | 0.29 | 0.13 |
| **377** | 0.11 | 0.06 | 10.00 | 10.00 | 0.13 | 1.11 | 0.31 | 0.29 | 0.29 | 0.13 |
| **376** | 0.11 | 0.06 | 10.00 | 10.00 | 0.13 | 1.12 | 0.31 | 0.29 | 0.29 | 0.13 |
| **375** | 0.11 | 0.06 | 10.00 | 10.00 | 0.14 | 1.13 | 0.31 | 0.29 | 0.29 | 0.14 |
| **374** | 0.11 | 0.07 | 10.00 | 10.00 | 0.14 | 1.13 | 0.31 | 0.29 | 0.29 | 0.14 |
| **373** | 0.11 | 0.07 | 10.00 | 10.00 | 0.14 | 1.13 | 0.31 | 0.29 | 0.29 | 0.14 |
| **372** | 0.11 | 0.07 | 10.00 | 10.00 | 0.14 | 1.13 | 0.31 | 0.29 | 0.29 | 0.14 |
| **371** | 0.11 | 0.07 | 10.00 | 10.00 | 0.14 | 1.13 | 0.31 | 0.29 | 0.29 | 0.14 |
| **370** | 0.11 | 0.07 | 10.00 | 10.00 | 0.14 | 1.13 | 0.31 | 0.29 | 0.29 | 0.14 |
| **369** | 0.11 | 0.08 | 10.00 | 10.00 | 0.14 | 1.13 | 0.31 | 0.29 | 0.29 | 0.14 |
| **368** | 0.12 | 0.08 | 10.00 | 10.00 | 0.15 | 1.16 | 0.31 | 0.30 | 0.30 | 0.15 |
| **367** | 0.11 | 0.08 | 10.00 | 10.00 | 0.14 | 1.12 | 0.31 | 0.29 | 0.29 | 0.14 |
| **366** | 0.11 | 0.08 | 10.00 | 10.00 | 0.14 | 1.11 | 0.31 | 0.30 | 0.30 | 0.14 |
| **365** | 0.11 | 0.09 | 10.00 | 10.00 | 0.14 | 1.11 | 0.31 | 0.30 | 0.30 | 0.14 |
| **364** | 0.11 | 0.10 | 10.00 | 10.00 | 0.15 | 1.11 | 0.31 | 0.30 | 0.30 | 0.15 |
| **363** | 0.11 | 0.10 | 10.00 | 10.00 | 0.15 | 1.12 | 0.32 | 0.30 | 0.30 | 0.15 |
| **362** | 0.11 | 0.11 | 10.00 | 10.00 | 0.15 | 1.12 | 0.31 | 0.30 | 0.30 | 0.15 |
| **361** | 0.12 | 0.12 | 10.00 | 10.00 | 0.15 | 1.12 | 0.32 | 0.30 | 0.30 | 0.15 |
| **360** | 0.12 | 0.13 | 10.00 | 10.00 | 0.15 | 1.12 | 0.32 | 0.30 | 0.30 | 0.15 |
| **359** | 0.12 | 0.15 | 10.00 | 10.00 | 0.15 | 1.12 | 0.32 | 0.30 | 0.30 | 0.15 |
| **358** | 0.12 | 0.16 | 10.00 | 10.00 | 0.15 | 1.12 | 0.32 | 0.30 | 0.30 | 0.15 |
| **357** | 0.11 | 0.18 | 10.00 | 10.00 | 0.15 | 1.10 | 0.32 | 0.30 | 0.30 | 0.15 |
| **356** | 0.12 | 0.21 | 3.55 | 10.00 | 0.16 | 1.09 | 0.32 | 0.30 | 0.30 | 0.16 |
| **355** | 0.12 | 0.23 | 2.90 | 10.00 | 0.16 | 1.11 | 0.32 | 0.30 | 0.30 | 0.16 |
| **354** | 0.12 | 0.27 | 2.63 | 10.00 | 0.16 | 1.11 | 0.32 | 0.30 | 0.30 | 0.16 |
| **353** | 0.12 | 0.30 | 2.15 | 10.00 | 0.16 | 1.10 | 0.32 | 0.30 | 0.30 | 0.16 |
| **352** | 0.12 | 0.35 | 2.33 | 10.00 | 0.16 | 1.10 | 0.32 | 0.30 | 0.30 | 0.16 |
| **351** | 0.12 | 0.41 | 2.21 | 10.00 | 0.16 | 1.10 | 0.32 | 0.30 | 0.30 | 0.16 |
| **350** | 0.12 | 0.46 | 2.09 | 10.00 | 0.16 | 1.09 | 0.32 | 0.30 | 0.30 | 0.16 |
