# Supplementary material for: A colorimetric assay for vanillin detection by determination of the luminescence of o-toluidine condensates
Source: PLoS One. 2018 Apr 20;13(4):e0194010. doi: 10.1371/journal.pone.0194010 (PMC5909897; doi:10.1371/journal.pone.0194010)
Supplement: S3 Table — Spectra of standard samples from 1 μg mL−1 to 100 μg mL−1 before heating. (DOCX) [file pone.0194010.s003.docx]

**S3 table. The UV-vis absorption curve data of Fig. 2 C.** Spectra of standard samples from 1 µg mL^−1^ to 100 µg mL^−1^ before heating.

| **Wavelength (nm)** | **The Absorbance of different** **vanillin concentration (µg/mL) before heating** | | | | | |
| --- | --- | --- | --- | --- | --- | --- |
|  | **100** | **75** | **50** | **25** | **1** | **Blank** |
| **400** | 0.34 | 0.27 | 0.27 | 0.26 | 0.26 | 0.13 |
| **399** | 0.35 | 0.28 | 0.28 | 0.27 | 0.27 | 0.13 |
| **398** | 0.35 | 0.29 | 0.28 | 0.28 | 0.27 | 0.14 |
| **397** | 0.36 | 0.29 | 0.29 | 0.29 | 0.28 | 0.15 |
| **396** | 0.37 | 0.30 | 0.30 | 0.30 | 0.29 | 0.16 |
| **395** | 0.38 | 0.31 | 0.31 | 0.31 | 0.30 | 0.17 |
| **394** | 0.39 | 0.32 | 0.32 | 0.32 | 0.31 | 0.18 |
| **393** | 0.41 | 0.33 | 0.33 | 0.33 | 0.32 | 0.19 |
| **392** | 0.42 | 0.34 | 0.34 | 0.34 | 0.33 | 0.20 |
| **391** | 0.43 | 0.35 | 0.35 | 0.35 | 0.34 | 0.21 |
| **390** | 0.44 | 0.36 | 0.36 | 0.36 | 0.35 | 0.22 |
| **389** | 0.45 | 0.37 | 0.37 | 0.37 | 0.36 | 0.23 |
| **388** | 0.46 | 0.38 | 0.38 | 0.38 | 0.37 | 0.24 |
| **387** | 0.46 | 0.39 | 0.39 | 0.39 | 0.38 | 0.25 |
| **386** | 0.47 | 0.40 | 0.40 | 0.40 | 0.39 | 0.26 |
| **385** | 0.48 | 0.41 | 0.41 | 0.41 | 0.40 | 0.27 |
| **384** | 0.49 | 0.42 | 0.42 | 0.42 | 0.41 | 0.28 |
| **383** | 0.50 | 0.43 | 0.43 | 0.43 | 0.42 | 0.29 |
| **382** | 0.51 | 0.44 | 0.44 | 0.44 | 0.43 | 0.30 |
| **381** | 0.52 | 0.45 | 0.45 | 0.44 | 0.43 | 0.31 |
| **380** | 0.53 | 0.45 | 0.46 | 0.45 | 0.44 | 0.32 |
| **379** | 0.54 | 0.46 | 0.47 | 0.46 | 0.45 | 0.32 |
| **378** | 0.54 | 0.47 | 0.48 | 0.47 | 0.46 | 0.33 |
| **377** | 0.55 | 0.48 | 0.48 | 0.48 | 0.46 | 0.34 |
| **376** | 0.55 | 0.48 | 0.49 | 0.48 | 0.47 | 0.35 |
| **375** | 0.56 | 0.49 | 0.49 | 0.49 | 0.48 | 0.35 |
| **374** | 0.56 | 0.50 | 0.50 | 0.50 | 0.48 | 0.36 |
| **373** | 0.56 | 0.50 | 0.51 | 0.50 | 0.49 | 0.37 |
| **372** | 0.57 | 0.51 | 0.51 | 0.51 | 0.50 | 0.37 |
| **371** | 0.57 | 0.51 | 0.52 | 0.52 | 0.50 | 0.38 |
| **370** | 0.58 | 0.52 | 0.52 | 0.52 | 0.50 | 0.38 |
| **369** | 0.58 | 0.52 | 0.53 | 0.52 | 0.51 | 0.38 |
| **368** | 0.58 | 0.52 | 0.53 | 0.53 | 0.51 | 0.39 |
| **367** | 0.58 | 0.52 | 0.53 | 0.53 | 0.51 | 0.39 |
| **366** | 0.58 | 0.52 | 0.53 | 0.53 | 0.51 | 0.39 |
| **365** | 0.58 | 0.53 | 0.54 | 0.53 | 0.52 | 0.39 |
| **364** | 0.58 | 0.53 | 0.54 | 0.53 | 0.52 | 0.39 |
| **363** | 0.58 | 0.53 | 0.54 | 0.53 | 0.52 | 0.39 |
| **362** | 0.58 | 0.53 | 0.54 | 0.53 | 0.52 | 0.39 |
| **361** | 0.58 | 0.53 | 0.54 | 0.53 | 0.52 | 0.39 |
| **360** | 0.58 | 0.53 | 0.53 | 0.53 | 0.52 | 0.39 |
| **359** | 0.57 | 0.53 | 0.53 | 0.53 | 0.51 | 0.39 |
| **358** | 0.57 | 0.52 | 0.53 | 0.52 | 0.51 | 0.39 |
| **357** | 0.57 | 0.52 | 0.53 | 0.52 | 0.51 | 0.38 |
| **356** | 0.56 | 0.52 | 0.53 | 0.52 | 0.50 | 0.38 |
| **355** | 0.56 | 0.51 | 0.52 | 0.51 | 0.50 | 0.37 |
| **354** | 0.55 | 0.51 | 0.52 | 0.51 | 0.50 | 0.37 |
| **353** | 0.55 | 0.51 | 0.51 | 0.51 | 0.49 | 0.37 |
| **352** | 0.55 | 0.50 | 0.51 | 0.50 | 0.49 | 0.36 |
| **351** | 0.54 | 0.50 | 0.50 | 0.50 | 0.48 | 0.36 |
| **350** | 0.54 | 0.49 | 0.50 | 0.49 | 0.48 | 0.35 |
