# Supplementary material for: A colorimetric assay for vanillin detection by determination of the luminescence of o-toluidine condensates
Source: PLoS One. 2018 Apr 20;13(4):e0194010. doi: 10.1371/journal.pone.0194010 (PMC5909897; doi:10.1371/journal.pone.0194010)
Supplement: S5 Table — Spectra of standard samples from 1 μg mL−1 to 100 μg mL−1 before heating. (DOCX) [file pone.0194010.s005.docx]

**S5 table. The UV-vis absorption curve data of Fig. 2 E.** Spectra of standard samples from 1 µg mL^−1^ to 100 µg mL^−1^ before heating.

| **Wavelength (nm)** | **The Absorbance of different** **vanillin concentration (µg/mL) before heating** | | | | | |
| --- | --- | --- | --- | --- | --- | --- |
|  | **100** | **75** | **50** | **25** | **1** | **Blank** |
| **560** | 0.22 | 0.19 | 0.18 | 0.18 | 0.19 | 0.03 |
| **559** | 0.23 | 0.20 | 0.19 | 0.19 | 0.19 | 0.03 |
| **558** | 0.23 | 0.20 | 0.19 | 0.19 | 0.20 | 0.03 |
| **557** | 0.24 | 0.21 | 0.20 | 0.19 | 0.20 | 0.03 |
| **556** | 0.24 | 0.21 | 0.20 | 0.20 | 0.20 | 0.03 |
| **555** | 0.24 | 0.21 | 0.21 | 0.20 | 0.20 | 0.03 |
| **554** | 0.25 | 0.22 | 0.21 | 0.21 | 0.21 | 0.03 |
| **553** | 0.25 | 0.22 | 0.22 | 0.21 | 0.21 | 0.03 |
| **552** | 0.26 | 0.23 | 0.22 | 0.21 | 0.21 | 0.03 |
| **551** | 0.26 | 0.23 | 0.22 | 0.22 | 0.22 | 0.03 |
| **550** | 0.27 | 0.24 | 0.23 | 0.22 | 0.22 | 0.03 |
| **549** | 0.27 | 0.24 | 0.23 | 0.23 | 0.22 | 0.03 |
| **548** | 0.28 | 0.25 | 0.24 | 0.23 | 0.23 | 0.03 |
| **547** | 0.28 | 0.25 | 0.24 | 0.23 | 0.23 | 0.03 |
| **546** | 0.29 | 0.26 | 0.25 | 0.24 | 0.23 | 0.03 |
| **545** | 0.29 | 0.26 | 0.25 | 0.24 | 0.24 | 0.03 |
| **544** | 0.30 | 0.26 | 0.26 | 0.25 | 0.24 | 0.03 |
| **543** | 0.30 | 0.27 | 0.26 | 0.25 | 0.24 | 0.03 |
| **542** | 0.30 | 0.27 | 0.26 | 0.25 | 0.24 | 0.03 |
| **541** | 0.31 | 0.27 | 0.27 | 0.25 | 0.24 | 0.03 |
| **540** | 0.31 | 0.28 | 0.27 | 0.26 | 0.25 | 0.03 |
| **539** | 0.31 | 0.28 | 0.27 | 0.26 | 0.25 | 0.03 |
| **538** | 0.31 | 0.28 | 0.27 | 0.26 | 0.25 | 0.03 |
| **537** | 0.32 | 0.28 | 0.28 | 0.26 | 0.25 | 0.02 |
| **536** | 0.32 | 0.29 | 0.28 | 0.27 | 0.25 | 0.02 |
| **535** | 0.32 | 0.29 | 0.28 | 0.27 | 0.25 | 0.02 |
| **534** | 0.32 | 0.29 | 0.28 | 0.27 | 0.25 | 0.02 |
| **533** | 0.32 | 0.29 | 0.28 | 0.27 | 0.25 | 0.02 |
| **532** | 0.32 | 0.29 | 0.28 | 0.27 | 0.25 | 0.02 |
| **531** | 0.32 | 0.29 | 0.28 | 0.27 | 0.25 | 0.02 |
| **530** | 0.32 | 0.29 | 0.28 | 0.27 | 0.25 | 0.02 |
| **529** | 0.32 | 0.29 | 0.28 | 0.27 | 0.25 | 0.02 |
| **528** | 0.32 | 0.29 | 0.28 | 0.26 | 0.25 | 0.02 |
| **527** | 0.32 | 0.29 | 0.28 | 0.26 | 0.25 | 0.02 |
| **526** | 0.32 | 0.28 | 0.28 | 0.26 | 0.25 | 0.02 |
| **525** | 0.31 | 0.28 | 0.27 | 0.26 | 0.25 | 0.02 |
| **524** | 0.31 | 0.28 | 0.27 | 0.26 | 0.25 | 0.02 |
| **523** | 0.31 | 0.28 | 0.27 | 0.26 | 0.24 | 0.02 |
| **522** | 0.31 | 0.28 | 0.27 | 0.26 | 0.24 | 0.02 |
| **521** | 0.31 | 0.28 | 0.27 | 0.25 | 0.24 | 0.02 |
| **520** | 0.30 | 0.27 | 0.26 | 0.25 | 0.24 | 0.02 |
| **519** | 0.30 | 0.27 | 0.26 | 0.25 | 0.24 | 0.01 |
| **518** | 0.30 | 0.27 | 0.26 | 0.25 | 0.24 | 0.01 |
| **517** | 0.30 | 0.27 | 0.26 | 0.25 | 0.23 | 0.01 |
| **516** | 0.30 | 0.26 | 0.26 | 0.25 | 0.23 | 0.01 |
| **515** | 0.29 | 0.26 | 0.25 | 0.24 | 0.23 | 0.01 |
| **514** | 0.29 | 0.26 | 0.25 | 0.24 | 0.23 | 0.01 |
| **513** | 0.29 | 0.26 | 0.25 | 0.24 | 0.23 | 0.01 |
| **512** | 0.29 | 0.25 | 0.25 | 0.24 | 0.23 | 0.01 |
| **511** | 0.28 | 0.25 | 0.24 | 0.23 | 0.22 | 0.01 |
| **510** | 0.28 | 0.25 | 0.24 | 0.23 | 0.22 | 0.01 |
