# Supplementary material for: A colorimetric assay for vanillin detection by determination of the luminescence of o-toluidine condensates
Source: PLoS One. 2018 Apr 20;13(4):e0194010. doi: 10.1371/journal.pone.0194010 (PMC5909897; doi:10.1371/journal.pone.0194010)
Supplement: S7 Table — The absorption spectra of the vanillin samples from 1 μg mL−1 to 500 μg mL−1. (DOCX) [file pone.0194010.s007.docx]

**S7 table. The UV-vis absorption curve data of Fig. 3.** The absorption spectra of the vanillin samples from 1 µg mL^−1^ to 500 µg mL^−1^.

| **Wavelength (nm)** | **The Absorbance of different** **vanillin concentration (µg/mL)** | | | | | | | | | |
| --- | --- | --- | --- | --- | --- | --- | --- | --- | --- | --- |
|  | **500** | **400** | **300** | **200** | **100** | **75** | **50** | **25** | **1** | **Blank** |
| **400** | 0.78 | 0.74 | 0.67 | 0.58 | 0.51 | 0.45 | 0.43 | 0.41 | 0.39 | 0.29 |
| **399** | 0.79 | 0.75 | 0.69 | 0.60 | 0.52 | 0.47 | 0.44 | 0.42 | 0.40 | 0.30 |
| **398** | 0.83 | 0.78 | 0.71 | 0.62 | 0.54 | 0.48 | 0.45 | 0.43 | 0.41 | 0.30 |
| **397** | 0.86 | 0.80 | 0.73 | 0.63 | 0.55 | 0.49 | 0.46 | 0.45 | 0.42 | 0.31 |
| **396** | 0.89 | 0.82 | 0.75 | 0.65 | 0.57 | 0.51 | 0.48 | 0.46 | 0.43 | 0.32 |
| **395** | 0.91 | 0.83 | 0.78 | 0.67 | 0.58 | 0.52 | 0.49 | 0.47 | 0.44 | 0.32 |
| **394** | 0.95 | 0.85 | 0.80 | 0.69 | 0.59 | 0.53 | 0.50 | 0.48 | 0.45 | 0.33 |
| **393** | 0.98 | 0.88 | 0.82 | 0.70 | 0.61 | 0.55 | 0.51 | 0.49 | 0.46 | 0.34 |
| **392** | 1.01 | 0.89 | 0.84 | 0.72 | 0.62 | 0.56 | 0.53 | 0.51 | 0.48 | 0.34 |
| **391** | 1.04 | 0.91 | 0.86 | 0.74 | 0.64 | 0.58 | 0.54 | 0.52 | 0.49 | 0.35 |
| **390** | 1.06 | 0.94 | 0.89 | 0.76 | 0.66 | 0.59 | 0.55 | 0.53 | 0.50 | 0.36 |
| **389** | 1.09 | 0.95 | 0.90 | 0.78 | 0.67 | 0.61 | 0.57 | 0.54 | 0.51 | 0.36 |
| **388** | 1.12 | 0.97 | 0.93 | 0.79 | 0.68 | 0.62 | 0.58 | 0.56 | 0.52 | 0.37 |
| **387** | 1.15 | 1.00 | 0.95 | 0.81 | 0.70 | 0.63 | 0.59 | 0.57 | 0.53 | 0.38 |
| **386** | 1.18 | 1.02 | 0.97 | 0.83 | 0.71 | 0.65 | 0.61 | 0.58 | 0.54 | 0.38 |
| **385** | 1.20 | 1.04 | 0.99 | 0.84 | 0.72 | 0.66 | 0.62 | 0.59 | 0.55 | 0.39 |
| **384** | 1.24 | 1.06 | 1.00 | 0.85 | 0.74 | 0.67 | 0.63 | 0.60 | 0.57 | 0.40 |
| **383** | 1.25 | 1.08 | 1.01 | 0.87 | 0.75 | 0.68 | 0.64 | 0.61 | 0.58 | 0.40 |
| **382** | 1.28 | 1.09 | 1.02 | 0.88 | 0.76 | 0.69 | 0.65 | 0.62 | 0.59 | 0.41 |
| **381** | 1.29 | 1.12 | 1.04 | 0.90 | 0.77 | 0.70 | 0.66 | 0.63 | 0.59 | 0.42 |
| **380** | 1.31 | 1.14 | 1.04 | 0.91 | 0.78 | 0.72 | 0.67 | 0.64 | 0.60 | 0.42 |
| **379** | 1.34 | 1.15 | 1.05 | 0.92 | 0.80 | 0.73 | 0.68 | 0.65 | 0.61 | 0.43 |
| **378** | 1.36 | 1.17 | 1.06 | 0.94 | 0.80 | 0.74 | 0.69 | 0.66 | 0.62 | 0.43 |
| **377** | 1.37 | 1.18 | 1.07 | 0.95 | 0.81 | 0.74 | 0.70 | 0.67 | 0.63 | 0.44 |
| **376** | 1.37 | 1.19 | 1.09 | 0.96 | 0.82 | 0.75 | 0.71 | 0.67 | 0.63 | 0.44 |
| **375** | 1.39 | 1.20 | 1.10 | 0.97 | 0.83 | 0.76 | 0.71 | 0.68 | 0.63 | 0.44 |
| **374** | 1.40 | 1.21 | 1.11 | 0.97 | 0.83 | 0.77 | 0.72 | 0.68 | 0.64 | 0.45 |
| **373** | 1.42 | 1.22 | 1.11 | 0.98 | 0.84 | 0.77 | 0.73 | 0.69 | 0.65 | 0.45 |
| **372** | 1.42 | 1.23 | 1.12 | 0.98 | 0.84 | 0.78 | 0.73 | 0.70 | 0.65 | 0.45 |
| **371** | 1.43 | 1.23 | 1.13 | 0.99 | 0.85 | 0.79 | 0.74 | 0.70 | 0.66 | 0.46 |
| **370** | 1.44 | 1.24 | 1.13 | 0.99 | 0.85 | 0.79 | 0.74 | 0.70 | 0.66 | 0.46 |
| **369** | 1.44 | 1.25 | 1.14 | 1.00 | 0.85 | 0.79 | 0.74 | 0.70 | 0.66 | 0.46 |
| **368** | 1.45 | 1.26 | 1.14 | 1.00 | 0.86 | 0.80 | 0.75 | 0.71 | 0.66 | 0.46 |
| **367** | 1.45 | 1.26 | 1.14 | 1.00 | 0.86 | 0.79 | 0.75 | 0.71 | 0.67 | 0.46 |
| **366** | 1.45 | 1.27 | 1.15 | 1.00 | 0.86 | 0.80 | 0.75 | 0.71 | 0.67 | 0.46 |
| **365** | 1.46 | 1.27 | 1.15 | 1.01 | 0.86 | 0.80 | 0.75 | 0.71 | 0.67 | 0.46 |
| **364** | 1.46 | 1.27 | 1.15 | 1.01 | 0.86 | 0.80 | 0.75 | 0.71 | 0.67 | 0.46 |
| **363** | 1.46 | 1.27 | 1.15 | 1.01 | 0.85 | 0.80 | 0.75 | 0.70 | 0.66 | 0.46 |
| **362** | 1.46 | 1.27 | 1.15 | 1.01 | 0.85 | 0.79 | 0.74 | 0.70 | 0.66 | 0.46 |
| **361** | 1.46 | 1.26 | 1.14 | 1.00 | 0.85 | 0.79 | 0.74 | 0.70 | 0.66 | 0.46 |
| **360** | 1.46 | 1.25 | 1.13 | 1.00 | 0.85 | 0.79 | 0.74 | 0.69 | 0.66 | 0.46 |
| **359** | 1.45 | 1.25 | 1.13 | 1.00 | 0.84 | 0.79 | 0.74 | 0.69 | 0.65 | 0.46 |
| **358** | 1.45 | 1.24 | 1.13 | 0.99 | 0.84 | 0.78 | 0.73 | 0.69 | 0.65 | 0.46 |
| **357** | 1.45 | 1.23 | 1.12 | 0.99 | 0.84 | 0.78 | 0.73 | 0.68 | 0.65 | 0.46 |
| **356** | 1.44 | 1.23 | 1.11 | 0.98 | 0.83 | 0.78 | 0.72 | 0.68 | 0.64 | 0.45 |
| **355** | 1.44 | 1.21 | 1.11 | 0.98 | 0.83 | 0.77 | 0.72 | 0.67 | 0.64 | 0.45 |
| **354** | 1.43 | 1.20 | 1.11 | 0.97 | 0.82 | 0.77 | 0.71 | 0.67 | 0.63 | 0.45 |
| **353** | 1.42 | 1.19 | 1.10 | 0.97 | 0.82 | 0.76 | 0.71 | 0.66 | 0.63 | 0.44 |
| **352** | 1.42 | 1.18 | 1.09 | 0.96 | 0.81 | 0.76 | 0.70 | 0.65 | 0.62 | 0.44 |
| **351** | 1.40 | 1.17 | 1.07 | 0.95 | 0.81 | 0.75 | 0.70 | 0.65 | 0.62 | 0.44 |
| **350** | 1.39 | 1.15 | 1.06 | 0.94 | 0.80 | 0.75 | 0.69 | 0.64 | 0.61 | 0.43 |
