# Supplementary material for: A colorimetric assay for vanillin detection by determination of the luminescence of o-toluidine condensates
Source: PLoS One. 2018 Apr 20;13(4):e0194010. doi: 10.1371/journal.pone.0194010 (PMC5909897; doi:10.1371/journal.pone.0194010)
Supplement: S8 Table — Milk powder samples compared with vanillin standard sample (50 μg mL-1). (DOCX) [file pone.0194010.s008.docx]

**S8 table. The UV-vis absorption curve data of Fig. 4.** Milk powder samples compared with vanillin standard sample (50 µg mL-1)

| **Wavelength (nm)** | **The Absorbance of milk powder samples** | | |
| --- | --- | --- | --- |
|  | **Powder sample 1** | **Powder sample 2** | **50 µg/mL** |
| **400** | 0.37766 | 0.43781 | 0.42658 |
| **399** | 0.38624 | 0.44337 | 0.43756 |
| **398** | 0.39561 | 0.44879 | 0.44853 |
| **397** | 0.40671 | 0.45457 | 0.46204 |
| **396** | 0.41485 | 0.46075 | 0.47573 |
| **395** | 0.42064 | 0.46673 | 0.48807 |
| **394** | 0.43121 | 0.47301 | 0.50063 |
| **393** | 0.43956 | 0.47929 | 0.51403 |
| **392** | 0.44853 | 0.48602 | 0.52744 |
| **391** | 0.46204 | 0.49285 | 0.54079 |
| **390** | 0.47573 | 0.49995 | 0.55413 |
| **389** | 0.48807 | 0.50674 | 0.56721 |
| **388** | 0.50063 | 0.51362 | 0.58063 |
| **387** | 0.51403 | 0.521 | 0.59345 |
| **386** | 0.52744 | 0.52847 | 0.6065 |
| **385** | 0.54079 | 0.53467 | 0.61938 |
| **384** | 0.55413 | 0.54117 | 0.63141 |
| **383** | 0.56721 | 0.54816 | 0.64298 |
| **382** | 0.58063 | 0.55496 | 0.65326 |
| **381** | 0.59345 | 0.55943 | 0.66347 |
| **380** | 0.6065 | 0.5681 | 0.67358 |
| **379** | 0.61938 | 0.57611 | 0.68288 |
| **378** | 0.63141 | 0.58373 | 0.69201 |
| **377** | 0.64298 | 0.59145 | 0.70022 |
| **376** | 0.65326 | 0.59968 | 0.70612 |
| **375** | 0.66347 | 0.60797 | 0.71139 |
| **374** | 0.67358 | 0.61417 | 0.72034 |
| **373** | 0.68288 | 0.62113 | 0.727 |
| **372** | 0.69201 | 0.62995 | 0.73094 |
| **371** | 0.70022 | 0.63753 | 0.73568 |
| **370** | 0.71012 | 0.64041 | 0.7392 |
| **369** | 0.71539 | 0.64101 | 0.74242 |
| **368** | 0.71934 | 0.64281 | 0.74527 |
| **367** | 0.71784 | 0.64279 | 0.74562 |
| **366** | 0.71569 | 0.64403 | 0.74596 |
| **365** | 0.71396 | 0.64279 | 0.746 |
| **364** | 0.70904 | 0.64062 | 0.74631 |
| **363** | 0.70604 | 0.63758 | 0.74516 |
| **362** | 0.70455 | 0.63433 | 0.74358 |
| **361** | 0.69852 | 0.63185 | 0.74195 |
| **360** | 0.69404 | 0.62843 | 0.73962 |
| **359** | 0.69315 | 0.62364 | 0.73647 |
| **358** | 0.68829 | 0.62243 | 0.72933 |
| **357** | 0.68364 | 0.61614 | 0.72733 |
| **356** | 0.67867 | 0.6116 | 0.7231 |
| **355** | 0.67301 | 0.6073 | 0.71865 |
| **354** | 0.66683 | 0.60151 | 0.71396 |
| **353** | 0.65715 | 0.60242 | 0.70904 |
| **352** | 0.65275 | 0.60881 | 0.70352 |
| **351** | 0.64612 | 0.61471 | 0.69543 |
| **350** | 0.63754 | 0.62189 | 0.69265 |
