# Supplementary material for: A colorimetric assay for vanillin detection by determination of the luminescence of o-toluidine condensates
Source: PLoS One. 2018 Apr 20;13(4):e0194010. doi: 10.1371/journal.pone.0194010 (PMC5909897; doi:10.1371/journal.pone.0194010)
Supplement: S9 Table — (DOCX) [file pone.0194010.s009.docx]

**S9 Table Measurements details data of vanillin spiked in milk powder samples (n = 6)**

| **Spiking level** | **Found** | **STDEVP** | **AVERAGE** | **RSD** |
| --- | --- | --- | --- | --- |
| **1000** | 1233 | 77.683117 | 1280 | 0.060693 |
| **1000** | 1235 |  |  |  |
| **1000** | 1423 |  |  |  |
| **1000** | 1190 |  |  |  |
| **1000** | 1336 |  |  |  |
| **1000** | 1263 |  |  |  |
| **500** | 554 | 25.865034 | 560 | 0.04619 |
| **500** | 578 |  |  |  |
| **500** | 594 |  |  |  |
| **500** | 524 |  |  |  |
| **500** | 531 |  |  |  |
| **500** | 579 |  |  |  |
| **10** | 9.92 | 0.7147435 | 9.825 | 0.07274 |
| **10** | 8.74 |  |  |  |
| **10** | 9.42 |  |  |  |
| **10** | 10.43 |  |  |  |
| **10** | 10.94 |  |  |  |
| **10** | 9.5 |  |  |  |
